# Neurological soft signs (NSS) and brain morphology in patients with chronic schizophrenia and healthy controls

**DOI:** 10.1371/journal.pone.0231669

**Published:** 2020-04-22

**Authors:** Christina J. Herold, Marco Essig, Johannes Schröder

**Affiliations:** 1 Department of General Psychiatry, Section of Geriatric Psychiatry, University of Heidelberg, Heidelberg, Germany; 2 Department of Radiology, University of Manitoba, Winnipeg, Canada; University of Toronto, CANADA

## Abstract

Subtle abnormalities in sensory integration, motor coordination and sequencing of complex motor acts or neurological soft signs (NSS) are characteristic phenomena in patients with schizophrenia at any stage of the illness. Previous MRI studies in schizophrenia found NSS to be associated with cortical, thalamic and cerebellar changes. Since these studies mainly focused on first-episode or recent onset schizophrenia, the cerebral correlates of NSS in chronic schizophrenia remained rather unclear. 49 middle-aged patients with chronic schizophrenia with a mean duration of illness of 20.3 ± 14.0 years and 29 healthy subjects matched for age and sex were included. NSS were examined on the Heidelberg Scale and correlated to grey matter (GM) by using whole brain high resolution magnetic resonance imaging (3 Tesla) with SPM12/CAT12 analyses. As expected, NSS in patients were significantly (p≤0.001) elevated in contrast to healthy controls, a finding, which not only applied to NSS total score, but also to the respective subscales “motor coordination”, “sensory integration”, “complex motor tasks”, “right/left and spatial orientation” and “hard signs”. Within the patient group NSS total scores were significantly correlated to reduced GM in right lingual gyrus, left parahippocampal gyrus, left superior temporal gyrus, left thalamus (medial dorsal nucleus) and left posterior lobe of the cerebellum (declive). Respective negative associations could also be revealed for the subscales “motor coordination”, “complex motor tasks” and “right/left and spatial orientation”. These findings remained significant after FWE-correction for multiple comparisons and were confirmed when years of education, chlorpromazine-equivalents or variables indicating the severity of psychopathology were introduced as additional covariates. According to our results lingual, parahippocampal, superior temporal, inferior and middle frontal gyri, thalamus and cerebellum have to be considered as important sites of NSS in chronic schizophrenia. That these findings only applied for patients but not healthy controls may indicate a different pathogenesis of NSS.

## Introduction

Neurological soft signs (NSS) refer to a broad range of discrete neurological abnormalities such as deficits in sensory integration, motor coordination and sequencing of complex motor acts [1, 2]. Elevated levels of NSS are frequently found in patients with schizophrenia at any stage of their illness as well as—although to a lower extent—in relatives of patients with schizophrenia [3–6]. NSS are associated with negative symptoms and cognitive impairments, severity and persistence of psychopathological symptoms and poor social functioning, but seem to be relatively independent of antipsychotic medication [3, 5, 7–10].

Structural and functional neuroimaging studies of NSS in schizophrenia identified the pre- and postcentral gyri, premotor area, cerebellum, middle and inferior frontal gyri, thalamus and basal ganglia, temporal and lingual gyri, inferior parietal lobule, insula, precuneus and occipital gyrus as important sites of NSS [11, 12]. Taken together these findings correspond strongly with the assumption of a disruption of the cortico-cerebellar-thalamic-cortical circuit in schizophrenia, as it was conceptualized as model of “cognitive dysmetria” by Andreasen et al. [13]. However, the majority of studies focused on patients with a first-episode to avoid potential influences of duration of illness and long-term neuroleptic treatment on brain structure [14–21].

Structural brain correlates of NSS in patients with chronic schizophrenia were scarcely investigated. One of the first studies [22] reported that NSS correlated not with cerebral ventricular size using computed tomography scans (CT) in a sample of 16 “middle aged” patients (mean age 44 years, mean duration of illness: 21 years). In a clinical CT-study of 50 patients with schizophrenia Schröder et al. [23] found NSS and global cerebral changes to be associated in patients with a chronic course of the disorder. Mohr et al. [24] examined 39 patients (17 of which belonged to a more chronic and disabled group) using CT; in this study NSS (Neurological Evaluation Scale, NES) were consistently related to the relative width of the third ventricle, the interhemispheric fissure and of the sulci laterales. These results remained significant after age and duration of hospitalization were partialled out. Recently, Kong and colleagues [25] examined the relationships between NSS and cortical thickness abnormalities in a group of patients (N = 18) with chronic schizophrenia (mean age: 54 years, mean duration of illness: 32 years). They found significant negative correlations between NSS and cortical thickness in prefrontal, inferior temporal, superior parietal, postcentral, and supramarginal cortices. Interestingly, this pattern of findings did not apply to the healthy controls (N = 20). In contrast, Kong et al. [25] found negative correlations between NSS and cortical thickness in anterior cingulate, pericalcarine and superior/middle temporal regions in their healthy control group. In younger healthy subjects (mean age: 27–30 years), higher rates of NSS were associated with a reduction of right middle frontal gyrus, inferior frontal gyrus, middle and superior temporal gyrus and anterior cingulate gyrus [21, 26].

A longitudinal analysis in 20 patients with first-episode schizophrenia (mean age: 26 years) of our workgroup [17] yielded a dichotomisation into patients with decreasing and patients with persistently increased NSS scores after a follow-up period of one year: While those with decreasing NSS scores showed only minor and localized changes within the left frontal lobe, cerebellum and cingulate gyrus, patients prone to persistently increased NSS scores showed pronounced grey matter (GM) reductions. These patients exhibited a more unfavourable course associated with GM decreases of the right sublobar claustrum, right cingulate gyrus, right cerebellum, left frontal lobe and left middle frontal gyrus.

Due to the above-mentioned limitations we examined a group of middle-aged patients with chronic schizophrenia and healthy controls to investigate the associations between NSS scores and structural brain correlates using a voxel-based morphometry approach. We expected higher NSS levels to be associated with reduced GM volume in cortical and subcortical sites in both groups, with different/diminished association patterns in healthy controls. More specifically, we hypothesized findings that parallel and extend those described in our former longitudinal study to further support the idea that persisting NSS refer to progressive cerebral changes.

## Material and methods

### Participants

Forty-nine patients with subchronic (n = 4) or chronic (n = 45) schizophrenia (N = 46) or schizoaffective disorder (N = 3) by DSM-III/DSM-IV criteria [27, 28] from the residential care St. Thomas e.V./Heidelberg (n = 28) and the Department of Psychiatry/University of Heidelberg (n = 21) and 29 healthy controls matched for age and sex were included. The healthy comparison subjects were recruited through newspaper advertisement. Sociodemographic characteristics of the respective groups are given in Table 1. As to be expected, healthy controls had significantly more years of education.

**Table 1:** Demographic characteristics of patients and healthy controls.

	Patients (n = 49)	Healthy controls (n = 29)	Main effects T-values_[df]_/χ^2^-values
Sex m/f, N Male, %	34/15 (69.4)	17/12 (58.6)	χ^2^ = 0.933; p = 0.334; φ = 0.109
Age, years	42.33 (13.93)	48.21 (13.56)	T_[76]_ = -1.820; p = 0.073
Education, years	12.39 (2.74)	13.88 (2.14)	T_[76]_ = -2.515; p = 0.014

Data are means (standard deviations), unless otherwise indicated

None of the participants had a lifetime history of neurological illness, head injury, substance dependency or an axis II disorder. Patients with extrapyramidal side effects, akathisia, parkinsonian signs and abnormal involuntary movements were excluded before study entry. Patients with late onset schizophrenia, i.e. with a manifestation of the disease after age 40 were not included [29].

The investigations were approved by the ethics committee of the Medical Faculty, Heidelberg University. Written informed consent was obtained from all participants in accordance with the Declaration of Helsinki after the procedures of the study had been fully explained.

### Clinical assessment

The severity of psychopathological symptoms was assessed with the Brief Psychiatric Rating Scale [BPRS, 30], the Scales for the Assessment of Positive and Negative Symptoms [SAPS and SANS, 31, 32] and the Apathy Evaluation Scale [AES, 33, 34]. Patients were treated with antipsychotic medication according to their psychiatrists’ choice.

NSS of patients and controls were evaluated using the Heidelberg Scale [2, 35], which consists of 16 items on five factors (“motor coordination”: Ozeretzki’s test, diadochokinesis, pronation/supination, finger-to-thumb opposition, speech and articulation; “sensory integration”: gait, tandem walking, two-point discrimination; “complex motor tasks”: finger-to-nose test, fist-edge-palm test; “right/left and spatial orientation”: right/left orientation, graphesthesia, face-hand test, stereognosis; “hard signs”: arm holding test, mirror movements). All items except for gait, tandem gait, Ozeretzki’s test, speech and articulation and right/left orientation were assessed separately for both, right and left sites respectively. Ratings were given on a 0–3 point scale (no/slight/moderate/marked abnormality). The psychometric properties of the Heidelberg Scale were well established in previous studies [2, 36]. The clinical assessments were performed by Christina J. Herold, Marc M. Lässer, and Dusan Hirjak, interrater agreements were ensured. Blinding with respect to diagnostic group was not possible, given a patient sample with severe mental illness.

### Image data acquisition and voxel-based morphometry

The magnetic resonance imaging (MRI) data were obtained at the German Cancer Research Centre on a 3.0 Tesla scanner (SIEMENS MAGNETOM TrioTim syngo MR B15). A high-resolution T1-weighted magnetization prepared rapid gradient echo (MP-RAGE) sequence was performed with the following acquisition parameters: 160 sagittal slices, voxel size = 1.0 x 1.0 x 1.0 mm, image matrix = 256 x 256, flip angle 9°, TR = 2300 ms, TE = 2.98 ms, TI = 900 ms.

After having visually checked all structural images for artefacts, the origin was manually set on the anterior commissure. We used the Statistical Parametric Mapping software (SPM12, v6685) implemented within Matlab R2015a to preprocess the MRI data (http://www.fil.ion.ucl.ac.uk/spm/software/). Voxel-based morphometry (VBM) analyses were conducted by using the Computational Anatomy Toolbox (CAT12 r963) with default parameters (http://www.neuro.uni-jena.de/cat/). Finally, the images were smoothed with an 8-mm full width at half-maximum (FWHM) Gaussian kernel.

### Statistical analyses

Group differences in demographic data were examined with independent two-tailed two-sample t-test and χ^2^ test using Statistical Package for the Social Sciences (IBM SPSS Statistics 23). Multivariate analysis of variance (MANOVA) was calculated to evaluate group differences on NSS total scores and the respective subscales. The potential associations between NSS scores and chlorpromazine (CPZ)-equivalents and psychopathological sumscores (SAPS, SANS BPRS, AES) were explored using bivariate correlations (Pearson's r). This approach was also used when calculating correlations between NSS scores and absolute GM volumes in the respective groups. In all analyses conventional significance levels (p<0.05) were applied.

To evaluate the relationships between GM and NSS scores we calculated voxel-wise regression analyses in patients and healthy controls separately, TIV (total intracranial volume) was used as covariate. The resulting T-maps were thresholded for a significance level of p<0.001 uncorrected and an extent threshold of k = 100 voxel. In a second step the family-wise error (FWE) correction was applied at cluster level to correct for multiple comparisons (p≤0.05).

After conversion of the resulting coordinates to Talairach space by using the icbm2tal transform [37, 38] implemented within GingerALE 2.3.6 [39–41], Talairach labels were generated by Talairach Client 2.4.3 [42, 43].

## Results

### Clinical characteristics

Clinical data of the patients are presented in detail in Table 2. Illness duration was 20 years on average and the majority of patients (57%) was institutionalized at the time of examination. All but one patient received antipsychotic medication.

**Table 2:** Clinical characteristics of the patients.

	Patients (n = 49)
Medication, mg CPZ-equivalents	638.37 (569.57)
Antipsychotic medication: AT, AT+T, T, no medication, N (%)	32/13/3/1 (65.3/26.5/6.1/2.0)
Additional antidepressive medication, N (%)	20 (40.8)
Additional benzodiazepines, N (%)	4 (8.3)
Illness duration, years	20.31 (14.00)
Age at illness onset, years	20.10 (6.03)
Hospitalized, N (%)	28 (57.1)
SAPS, Sum Score	14.67 (14.83)
SANS, Sum Score	26.29 (18.35)
BPRS, Sum Score	38.08 (9.66)
*BPRS—Anxiety/Depression*	10.20 (4.55)
*BPRS—Anergia*	9.94 (4.45)
*BPRS—Thought disturbance*	8.53 (3.85)
*BPRS—Activity*	4.37 (1.87)
*BPRS—Hostility/Suspiciousness*	5.04 (2.79)
AES, Sum Score	26.67 (11.96)

Data are means (standard deviations), unless otherwise indicated

*AES* Apathy Evaluation Scale; *AT* atypical antipsychotics; *AT+T* atypical and typical antipsychotics; *BPRS* Brief Psychiatric Rating Scale; *CPZ* chlorpromazine; *SANS* Scale for the Assessment of Negative Symptoms; *SAPS* Scale for the Assessment of Positive Symptoms; *T* typical antipsychotics

The ANOVA calculated to compare NSS scores between patients and controls yielded a significant main effect for “group” (F_[6,71]_ = 5.380, p<0.001, η^2^ = 0.313), indicating significantly increased NSS in patients in contrast to healthy controls. The respective results for NSS total scores and the five subscales are given in Table 3.

**Table 3:** Results of group comparisons on NSS.

	Patients	Healthy controls	Main effects, F-values_[df]_, effect size η^2^
NSS total score	16.90 (12.37)	3.79 (3.29)	F_[1,76]_ = 31.094*** η^2^ = 0.290
*motor coordination*	6.67 (5.60)	1.69 (1.44)	F_[1,76]_ = 21.975*** η^2^ = 0.224
*sensory integration*	2.51 (2.12)	0.52 (0.79)	F_[1,76]_ = 23.553*** η^2^ = 0.237
*complex motor tasks*	2.82 (2.51)	0.66 (1.17)	F_[1,76]_ = 18.914*** η^2^ = 0.199
*right/left and spatial orientation*	3.47 (3.39)	0.48 (0.95)	F_[1,76]_ = 21.388*** η^2^ = 0.220
*hard signs*	1.61 (1.69)	0.45 (0.74)	F_[1,76]_ = 12.276*** η^2^ = 0.139

Data are means (standard deviations)

***p≤0.001

No significant correlations between CPZ-equivalents and NSS total scores or the respective subscales (p>0.20) arose. The sumscores of the psychopathological instruments SAPS, SANS BPRS were not significantly correlated to NSS sumcores (p>0.30), while AES sumcores were significantly associated to NSS total scores (r = 0.288, p = 0.045).

### Volumetric correlates of NSS

When calculating Pearson's correlation coefficients between absolute GM volumes and NSS scores we obtained significant negative associations for all but the subscales “complex motor tasks” and “hard signs” with -0.25>r>-0.45 (0.05>p>0.001) in the patient group (Table 4). In the controls a significant inverse correlation can be reported for the subscale “motor coordination” only (r = -0.44, p = 0.018).

**Table 4:** Correlations between NSS scores and absolute GM volumes in patients with chronic schizophrenia.

	GM (r/p-values)
NSS total score	-.400 (0.004)
*motor coordination*	-.388 (0.006)
*sensory integration*	-.284 (0.048)
*complex motor tasks*	-.245 (0.090)
*right/left and spatial orientation*	-.441 (0.002)
*hard signs*	-.029 (0.845)

The results of the volumetric correlates of NSS in patients are given in Table 5. In the patient group, higher NSS total scores were significantly correlated with decreased volume of right lingual (BA 18) as well as left parahippocampal gyrus (BA 28), left superior temporal gyrus (BA 38), left thalamus (medial dorsal nucleus) and left cerebellum (declive). Similar associations applied to the NSS subscale “motor coordination”. The subscale “complex motor tasks” showed a significant inverse association with left inferior frontal gyrus (BA 47). Increased scores on the subscale “right/left and spatial orientation” were correlated with GM loss in right middle frontal (BA 9), left lingual (BA 18) and left parahippocampal gyrus (BA 36). These associations remained significant after a stringent family-wise error correction for multiple comparisons (p<0.05). With respect to the subscale “sensory integration” none of the results survived the correction for multiple comparisons, in case of the subscale “hard signs” no significant correlations emerged.

**Table 5:** Anatomical structures showing significant inverse correlations between GM and NSS in patients with chronic schizophrenia.

	Anatomical structures	Cluster size (voxel)	T-value	Peak coordinates^a^ (x, y, z)
NSS total score				
	Right Occipital Lobe, Lingual Gyrus (BA 18)	1221	5.02	11, -90, -12
	Left Limbic Lobe, Parahippocampal Gyrus (BA 28)	1708	4.78	-26, -21, -24
	Left Temporal Lobe, Superior Temporal Gyrus (BA 38)	1158	4.77	-42, 9, -35
	Left Thalamus, Medial Dorsal Nucleus	1127	4.77	0, -15, 8
	Left Cerebellum, Posterior Lobe, Declive	1224	4.55	-35, -59, -8
*motor coordination*	Left Limbic Lobe, Parahippocampal Gyrus (BA 35)	2316	5.31	-27, -20, -26
	Right Cerebellum, Posterior Lobe, Declive	1054	4.92	23, -86, -14
	Left Cerebellum, Anterior Lobe, Culmen	1786	4.66	-54, -54, -30
	Right Limbic Lobe, Parahippocampal Gyrus (BA 28)	990	4.59	27, -17, -26
	Left Thalamus	1020	4.37	0, -14, 8
*complex motor tasks*	Left Frontal Lobe, Inferior Frontal Gyrus (BA 47)	1040	5.10	-50, 21, -9
*right/left and spatial orientation*	Right Frontal Lobe, Middle Frontal Gyrus (BA 9)	970	5.35	30, 42, 30
	Left Occipital Lobe, Lingual Gyrus (BA 18)	1270	4.96	2, -86, 2
	Left Limbic Lobe, Parahippocampal Gyrus (BA 36)	2265	4.76	-24, -33, -17

BA = Brodmann area; extent threshold k = 100 voxel, all regions survived a threshold of p≤0.05 (FWE-corrected)

^a^ Talairach coordinates

In a further step the correlations between GM and NSS scores controlled for CPZ-equivalents or BPRS/SAPS/SANS sum scores, respectively, were calculated. These results were very similar to those described above without these variables as additional covariates. The same applied for the respective results with years of education as covariate.

The tests for possible positive correlations between GM and NSS yielded in case of the subscale “right/left and spatial orientation” a significant correlation with right posterior lobe of the cerebellum (inferior semilunar lobule), k = 250 voxels, T = 3.91, 23/-87/-39. However, this result did not survive the FWE-correction for multiple comparisons.

None of the clinical parameters (sumscores of BPRS, SAPS, SANS, AES, CPZ-equivalents) showed associations with GM, that survived the correction for multiple comparisons. Similarly, the absolute GM volumes as calculated by CAT12 did not significantly correlate with these clinical parameters (p>0.08).

In the control group VBM revealed that higher NSS total scores were significantly correlated with volume loss in right pre- and postcentral gyrus, middle temporal and right superior temporal gyrus, however, these associations did not survive the FWE-correction for multiple comparisons. This was the case for the subscale “motor coordination”, which correlated significantly with reduced volume of right frontal lobe, as well as the subscale “sensory integration”, which showed significant inverse correlations with left cingulate gyrus (BA 32), left cuneus (BA 19) and right postcentral gyrus (BA 3) (see Table 6). None of the positive correlations survived the correction for multiple comparisons. These results remained the same when years of education were used as additional covariate.

**Table 6:** Anatomical structures showing significant inverse correlations between GM and NSS in healthy controls.

	Anatomical structures	Cluster size (voxel)	T-value	Peak coordinates^a^ (x, y, z)
*motor coordination*				
	Right Frontal Lobe, Sub-Gyral	686	4.90	36, 53, -9
*sensory integration*				
	Left Limbic Lobe, Cingulate Gyrus (BA 32)	805	6.03	2, 39, 21
	Left Occipital Lobe, Cuneus (BA 19)	1563	5.66	3, -81, 36
	Right Parietal Lobe, Postcentral Gyrus (BA 3)	872	5.03	29, -29, 59

BA = Brodmann area; extent threshold k = 100 voxel, all regions survived a threshold of p≤0.05 (FWE-corrected)

^a^ Talairach coordinates

Figures of the respective results can be found in the supporting information section of this article (S1 Fig).

## Discussion

This study investigated the associations between GM and NSS scores in a sample of middle-aged patients with chronic schizophrenia and healthy controls. Three main findings emerged: (1) a confirmation of significantly elevated NSS scores in patients, which (2) were significantly correlated to volume loss in bilateral lingual, parahippocampal, left superior temporal, left inferior and right middle frontal gyri, thalamus and cerebellum. (3) By contrast, in the healthy controls NSS showed significant negative correlations with volumes of left cingulate gyrus, cuneus, right frontal lobe and right postcentral gyrus.

According to the literature NSS are structurally associated with morphological alterations of pre- and postcentral gyri, premotor area, cerebellum, middle and inferior frontal gyri, thalamus and basal ganglia, temporal and lingual gyri, inferior parietal lobule, insula, precuneus and occipital gyrus in younger patient samples [for review see: 11, 12]. In addition, in middle-aged patients with chronic schizophrenia significant associations between NSS scores (Heidelberg Scale) and reduced cortical thickness in prefrontal, inferior temporal, superior parietal, postcentral and supramarginal cortices were demonstrated [25]. These findings parallel those of the present study and reflect the model of “cognitive dysmetria” developed by Andreasen et al. [13].

Moreover, corresponding to our own longitudinal results [17] we found in the present study GM in frontal and cerebellar regions to be negatively correlated with NSS scores. These finding confirms at least partly our hypothesis of associations between persistent NSS in patients with a chronic course of the disease and progressive cerebral changes. Discrepancies referring to the NSS-related brain regions can be explained by the different samples, with first-episode schizophrenia patients in the longitudinal approach.

In contrast to other studies we could not find any significant associations between NSS and basal ganglia. Given a sample of patients with chronic schizophrenia potential influences of antipsychotic medication on brain structure have to be considered, and may primarily affect basal ganglia volume, with typical antipsychotics increasing their volume [44–46]. However, it is generally accepted that NSS are not the sequellae of neuroleptic treatment. Rather, one study which investigated NSS with respect to the d2-dopamine receptor density in first-episode patients receiving a standard treatment with benperidol by using IBZM SPECT found NSS not to be associated with poor treatment response and receptor upregulation [47]. Therefore, an association between NSS and basal ganglia volume in patients with chronic schizophrenia may be attenuated.

Moreover, as in the present study we could not find significant correlations between antipsychotic dosage and NSS scores in a recent publication with 90 chronic schizophrenic patients [10]. Additionally, patients with extrapyramidal side effects, tardive dyskinesia, parkinsonian signs and abnormal involuntary movements were not included in the present study. While not all patients in our study received exclusively atypical antipsychotic medication, Jahn and colleagues [48] described earlier that NSS were not significantly different between patients receiving clozapine and conventional neuroleptics, thus concluding that NSS in schizophrenic psychoses seem to be relatively independent of antipsychotic side effects, but instead were associated with severity and persistence of psychopathological symptoms. The independency of NSS of the effects of medication is also supported by findings of elevated NSS in neuroleptic-naïve patients and/or with first-episode of schizophrenia [8, 49], and even in high risk subjects [50] or relatives of people with schizophrenia [4, 6] and by reports that described decreasing NSS scores under antipsychotic treatment in (drug-naïve) patients [2, 47, 51, 52].

In general, a heterogeneous picture was revealed in several overviews, which focused on possible structural brain changes associated with antipsychotic treatment in schizophrenia: Authors emphasized not only inconsistencies among the results, but also the question whether brain structural changes in patients with schizophrenia are due to the use of antipsychotic medication or intrinsic pathogenetic processes of the disorder itself or a combination of both [53–56]. Moreover, from a clinical standpoint physical diseases, reduced physical exercise and cognitive stimulation may also affect brain structure, particularly in a sample of patients with a chronic course of the disorder [57–59]. Therefore, we cannot exclude potential medication effects on brain structure, although significant interactions between GM and CPZ-equivalents did not arise in our sample and our results remained consistent when this variable was introduced as additional covariate.

Given potential associations of psychopathology (BPRS, SAPS, SANS, AES) and brain structure [60–62] this can be excluded in the present study as none of the respective associations with GM survived the correction for multiple comparisons. The inclusion of sumscores of psychopathological severity variables as additional covariates in our voxel-wise regression analyses yielded consistent results. Moreover, none of the psychopathological indices correlated significantly to NSS total scores, except AES sumcores, which can be interpreted as reflection of the chronicity of the disorder in our sample. As apathy can arise in various severe neuropsychiatric disorders including schizophrenia, mild cognitive impairment and Alzheimer’s disease [34, 63] it can be seen as a much broader concept than negative symptoms [10].

More specifically, the subscale “motor coordination” in our study was significantly correlated to bilateral volume losses in parahippocampal gyri, cerebellum (declive, culmen) and left thalamus. The subscale “complex motor tasks” showed a significant inverse correlation with left inferior frontal gyrus and the subscale “right/left and spatial orientation” had significant negative correlations with right middle frontal gyrus, left lingual and parahippocampal gyrus.

The frontal cortex is not only engaged in executive functions but also in planning, generating and monitoring sequences of actions [64, 65], as reflected by the present correlations between inferior and middle frontal gyri and the NSS subscales “complex motor tasks” and “right/left and spatial orientation” (“motor coordination” in healthy controls).

Our finding of a consistent association between NSS and reduced parahippocampal volumes might be interpreted with its role in memory functions, which are required for learning new motor sequences as in NSS assessment [66]. The involvement of the temporal gyrus can be interpreted with its contribution to auditory and language processes, visual information, visual recognition and audiovisual integration [67]. Interestingly, it has been shown that learning motor sequences requires the involvement of bilateral superior and anterior prefrontal cortex, right superior temporal cortex and left cerebellum [68]. Along with this, we recently described that NSS reflect a rather wide range of cognitive deficits in schizophrenia [69], a finding that may additionally explain the involvement of frontal and temporal sites.

The finding of cerebellar involvement in the performance of “motor coordination” is plausible insofar as it underlines its important role in motor control, motor learning and motor coordination [70, 71] and, moreover, its role in schizophrenia has been highlighted by Andreasen’s hypothesis of “cognitive dysmetria” [72].

Involvement of the thalamus seems to be self-evident, because it is generally considered to be a relay centre in coordination of motor activity by basal ganglia and cerebellar projections [73] and to mediate sensory perception [74]. More specifically, thalamic subregions have been shown to play important roles in visuospatial perception and attention as well as in action control as part of a circuit including the basal ganglia, motor, premotor and prefrontal cortices [75].

With respect to occipital contribution (lingual gyrus, cuneus) to NSS of the subscale “right/left and spatial orientation” (“sensory integration” in controls) imagery processes during the anticipation and interpretation of limb positions have to be considered, as these regions (BA 18, 19) are the site of the secondary and tertiary visual cortices [76].

In our control group of 29 middle-aged healthy subjects the correlations between NSS scores and GM were restricted to the subscales “motor coordination” and “sensory integration”, with areas (frontal lobe, cingulate gyrus, cuneus, postcentral gyrus) only partially overlapping to those found in the patient group. Similarly, NSS in younger healthy probands were also reported to correlate with frontal and cingulate regions [21, 26], but not cerebellar volume [77]. The cingulum is discussed to be involved in imagery of limb position, language and motor tasks requiring somatosensory control [78]. The postcentral gyrus plays as location of the primary somatosensory cortex a critical role in processing of somatosensory input and therefore contributes to the integration of sensory and motor signals [79].

The diminished and different correlation patterns between GM and NSS scores in our control group in contrast to the patients can be explained with their attenuated variance of NSS, which are rather low and stable over time. It has been shown that NSS in healthy controls may refer to age, level of education or less developed skills [10, 21, 36, 80]. Further evidence that NSS in patients with schizophrenia and healthy controls may be based on different pathogenetic factors came from the Northern Finland 1966 general population birth cohort study: Infant motor development was rated at age 1 year and related to cerebral changes (MRI) at age 33–35 years. Patients with schizophrenia did not show normative association patterns between frontocerebellar structure and infant motor development as healthy controls, which lead to the conclusion that NSS in schizophrenia are only partly based on a delayed infant motor development [81]. Moreover, NSS in healthy controls increase according to the physiological aging process, however this effect is reduced in contrast to patients with schizophrenia [10, 80, 82]. As this process only involves minor changes and differs with regard to individual predispositions or selective vulnerabilities no systematic associations between NSS scores and GM volumes might be detectable in healthy controls.

Our control group had significantly more years of education, which conforms to the fact that the disease with its onset in early adulthood often prevents patients from achieving higher levels of education. A significant influence of education on the reported associations between NSS scores and GM can be ruled out in both groups, as the respective analyses yielded consistent results when years of education were used as additional covariate.

The strengths of this study include the choice of a sample of chronically ill patients with an illness duration of two decades on average, who are strongly disabled reflected by about 60% living hospitalized. This patient group is considerably underrepresented as the majority of studies about structural brain correlates of NSS focused on patients with first-episode or recent-onset schizophrenia [12]. Moreover, due to our focus on patients with chronic schizophrenia the influence of varying courses of the disease can be excluded [17, 36]. Of course, factors as duration of illness and/or age may not only be associated with brain structure, but also with chronicity of psychosis and therefore a correction of these highly intercorrelated variables could mask important disease-related structural brain changes.

Paralleling previous results of a longitudinal study of our group [17] we confirmed associations between NSS and GM reductions in frontal and cerebellar sites. Therefore, the finding of progressive cerebral changes in first-episode schizophrenia that were related to persisting NSS scores could be strengthened and extended to a group of chronically ill patients. Specific vulnerable brain regions or characteristics of the disease itself may contribute to a chronic, unfavourable course.

As stated earlier, examiners were aware of patients’ symptoms and histories and therefore blinding with respect to diagnostic groups was not given, but, irrespective of that, this was not possible as a patient sample with severe mental illness was examined.

In conclusion, our findings demonstrate that NSS in patients with chronic schizophrenia are associated with volume loss in lingual, parahippocampal, superior temporal, inferior and middle frontal gyri, thalamus and cerebellum. These associations were not accounted for by CPZ-equivalents or severity of psychopathology and only applied for patients but not healthy controls, therefore indicating a different pathogenesis of NSS. Moreover, our results supported the assumption of progressive cerebral changes related to persisting NSS scores in patients with an unfavourable course of the disorder.

## Supporting information

S1 Fig Overview: Inverse correlations between GM volumes and NSS scores in patients with chronic schizophrenia and healthy controls. (TIF)
