# Supplementary figures and images for: Neurological soft signs (NSS) and brain morphology in patients with chronic schizophrenia and healthy controls
Source: PLoS One. 2020 Apr 22;15(4):e0231669. doi: 10.1371/journal.pone.0231669 (PMC7176089; doi:10.1371/journal.pone.0231669)

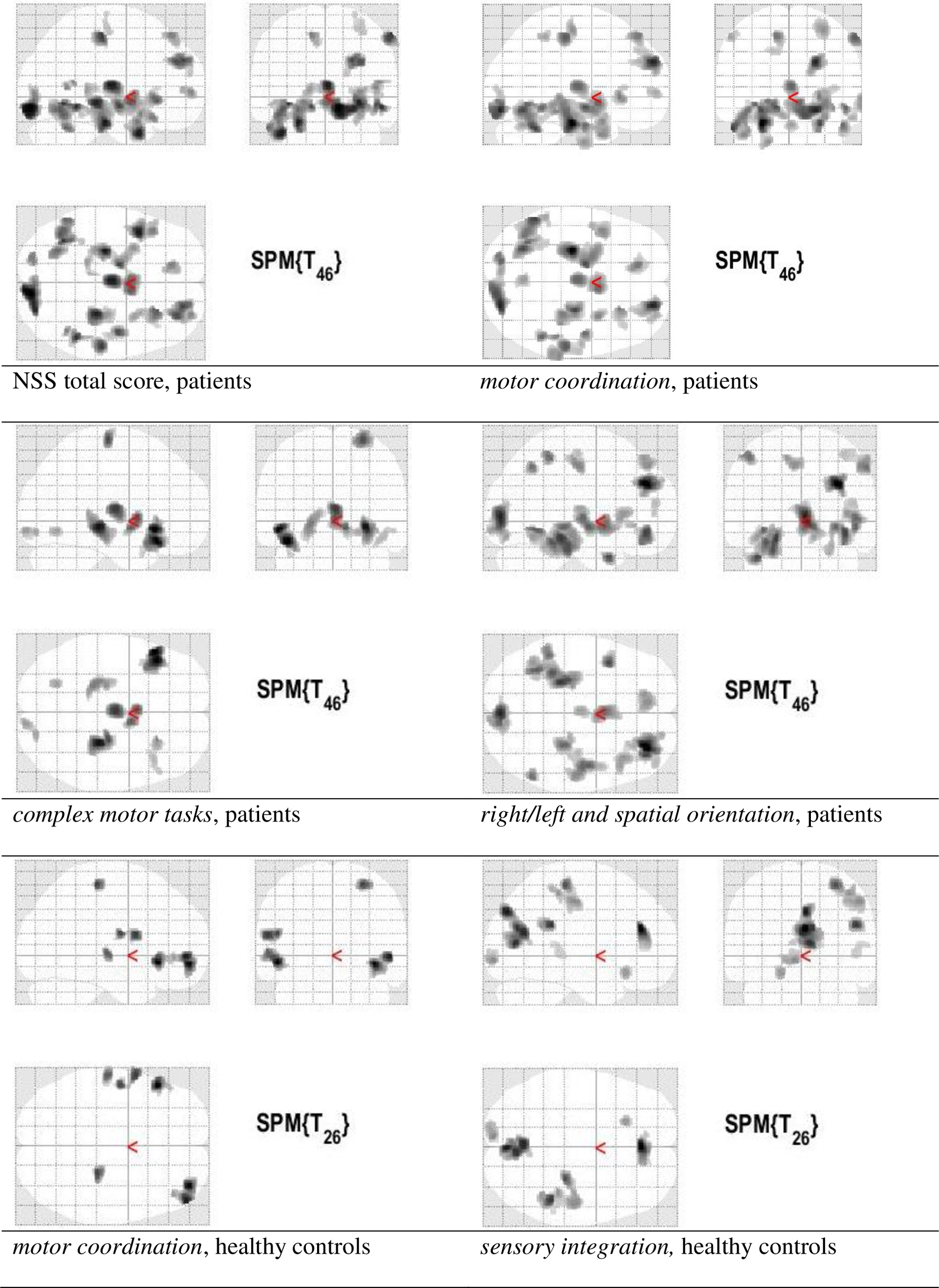

Supplement: S1 Fig — (TIF) [file pone.0231669.s001.tif]
